# Pathogen Profiles and Antimicrobial Resistance Patterns of Neonatal Sepsis in the Gulf Cooperation Council: A Systematic Review

**DOI:** 10.3390/children12111475

**Published:** 2025-11-01

**Authors:** Razique Anwer, Hassan Al-shehri, Musab Alsulami, Ziyad Alsulami, Faisal Alzkari, Nawaf Alshaalan, Nawaf Almutairi, Abdullah Saleh Albalawi, Khalid Alshammari, Abdulelah F. Alshehri, Nawaf Alzahrani, Ibrahim A. Alamer, Albaraa Alotaibi, Meshal Alzakari

**Affiliations:** 1 Department of Pathology, College of Medicine, Imam Mohammad Ibn Saud Islamic University (IMSIU), Riyadh 13317-4233, Saudi Arabia; 2 Department of Pediatrics, College of Medicine, Imam Mohammad Ibn Saud Islamic University (IMSIU), Riyadh 13317-4233, Saudi Arabia; 3 College of Medicine, Imam Mohammad Ibn Saud Islamic University (IMSIU), Riyadh 13317-4233, Saudi Arabia43014793@sm.imamu.edu.sa (Z.A.); 442019422@sm.imamu.edu.sa (N.A.); 442018747@sm.imamu.edu.sa (A.S.A.); 441017536@sm.imamu.edu.sa (M.A.)

**Keywords:** neonatal sepsis, pathogens, antibiotic resistance, Gulf Cooperation Council (GCC)

## Abstract

Background: Neonatal sepsis (NS) is a life-threatening condition in newborns, which is an infectious process with a systemic inflammatory reaction to bacterial, viral, or fungal infection acquired in the first 28 days of life. Methods: This study examines the major pathogens causing neonatal sepsis in the Gulf Cooperation Council (GCC) and their resistance patterns to antimicrobial agents. We utilized the Preferred Reporting Items for Systematic Reviews and Meta-Analyses (PRISMA) guidelines to develop this systematic review to follow a systematic and transparent process. The comprehensive literature review was done in several national and global databases, which include PubMed, Scopus, Google Scholar, Embase, and Cochrane Library. The key words inserted in the search strategy were “neonatal sepsis,” “late-onset sepsis,” “early-onset sepsis,” and “Gulf Cooperation Council (GCC),” and the keywords of antimicrobial resistance and pathogens were used: “antimicrobial drug resistance” and “pathogens.” Only articles published from January 1983 to January 2025 were included for screening. Results: The final count of the articles that fit the inclusion criteria is 54, and 5177 neonatal sepsis cases’ data have been identified. The most common pathogens were coagulase-negative staphylococci (CoNS) and *Klebsiella* spp., which caused 17.4 percent (901 cases) and 15.9 percent (825 cases) of neonatal sepsis, respectively. Coagulase-negative staphylococci (CoNS) were the most resistant, especially to oxacillin and erythromycin. The most isolated pathogens among *Gram-negative* spp. were *Klebsiella* spp., which showed a resistance to ampicillin, amoxicillin, and ceftriaxone. Conclusions: The bacterial isolates had a diversity of antimicrobial resistance, stressing the necessity of continuous hospital surveillance. Sophisticated diagnostic methods and well-designed research are necessary, especially in areas characterized by high rates of neonatal mortality, to determine the prevalence of neonatal sepsis, risk factors, and clinical outcomes.

## 1. Introduction

Neonatal sepsis remains one of the leading causes of infant mortality worldwide, with features indicative of a systemic inflammatory response to infection exhibiting non-specific and varied clinical signs [1,2]. This arises due to bacterial, viral, or fungal infections and can lead to multi-organ failure and death if not treated, usually within the first 28 days of life. The development of neonatal sepsis involves a multifaceted process that revolves around an immature immune system interacting with pathogens and the body’s defense mechanisms. Diagnosing sepsis, alongside identifying the microbial agents that cause bloodstream infections and their susceptibility to antibiotics, plays a crucial role in management. These two steps are essential to the management of sepsis, as they have a direct impact on the success of the therapy, length of hospitalization, and additional medical expenses.

Clinically, neonatal sepsis is divided into early-onset sepsis (EOS) and late-onset sepsis (LOS), each exhibiting distinct risk factors, causative agents, and clinical outcomes [3]. This classification provides insight into the variability in etiology, clinical outcomes, and therapeutic approaches associated with neonatal sepsis. EOS is identified within the first 72 h of life; causes of EOS are often linked to vertical transmission during delivery, whereas LOS is often associated with contamination from the surroundings [4]. EOS seems to have a higher mortality risk compared to LOS.

Neonatal sepsis is still a global health challenge, with incidence rates of 0.5–1.5 per 1000 live births in developed nations to as high as 5 per 1000 in low- and middle-income countries [5]. Multiparity and Cesarean Section (CS) delivery are key maternal risk factors for both EOS and LOS. Maternal genital and gastrointestinal tract flora may result in ascending bacterial infection in the uterine cavity, causing postnatal infections [6]. In developed countries, the common pathogens of early-onset neonatal sepsis are Group B Streptococcus (43–58%), *Escherichia coli* (18–29%), and other Gram-negative bacteria (7–8%). Likewise, in late-onset neonatal sepsis, the common pathogens are Coagulase-Negative Staphylococcus (39–54%), *E. coli* (5–13%), *Staphylococcus aureus* (6–18%), and Klebsiella (4–9%) [7,8,9].

Saudi Arabia’s healthcare system is considered one of the most developed in the GCC region, with well-equipped Neonatal Intensive Care Units (NICUs) providing special care for critically ill newborns. Despite these advancements, there is a gap in the understanding of the incidence, epidemiology, clinical features, and outcomes of neonatal sepsis in Saudi Arabia and neighboring GCC countries. There is insufficient data regarding the incidence, diagnosis, causative pathogens, antimicrobial resistance, and long-term prognosis in affected neonates, which calls for further investigation. The research conducted by Alalmaei and Alqahtani [10] and Tosson and Speer [11] specifically focuses on further investigating the absence of data surrounding neonatal sepsis in Saudi Arabia and the Gulf region. These gaps are essential to improve the protocol of neonatal care and advance the region’s healthcare services by enhancing the strategies for early detection, treatment, and, subsequently, reducing morbidity and mortality rates.

In addition to bacterial causes, fungal pathogens—particularly Candida species—are increasingly recognized as important contributors to neonatal sepsis, especially among premature and low-birth-weight infants. Candida infections can lead to severe bloodstream infections with high mortality if not detected early. Of particular concern is Candida auris, an emerging multidrug-resistant yeast reported globally and in several Middle Eastern countries [10]. It poses a major challenge due to its persistence in hospital environments, resistance to multiple antifungal agents, and potential for neonatal intensive care unit outbreaks, emphasizing the need for continuous regional surveillance and strict infection control measures. In this systematic review and meta-analysis, we conducted a comprehensive search for all available relevant studies aimed at isolating the major bacterial pathogens associated with neonatal sepsis in the Gulf Cooperation Council (GCC) region and studying their susceptibility to various antimicrobial agents to enhance strategies for infection control and optimal antimicrobial therapy in neonatal care.

## 2. Materials and Methods

### 2.1. Protocol Development and Search Strategy

We followed the Preferred Reporting Items for Systematic Reviews and Meta-Analyses (PRISMA) guidelines to ensure methodological rigor and transparency. This review is registered with the International Prospective Register of Systematic Reviews (PROSPERO) with the following register ID: CRD42024603984.

A comprehensive literature search was conducted across several databases, including PubMed, Scopus, Google Scholar, Embase, and the Cochrane Library. The search strategy incorporated key terms such as “neonatal sepsis,” “Late-Onset Sepsis,” “Early-Onset Sepsis,” and “Gulf Cooperation Council (GCC),” in conjunction with terms related to antimicrobial resistance and pathogens, including “Antimicrobial Drug Resistance” and “pathogens.”

### 2.2. Eligibility Criteria

The inclusion criteria were pre-defined to select relevant studies. Observational studies providing full-text information on neonatal sepsis, published between January 1985 to January 2025 and conducted in GCC countries (Saudi Arabia, UAE, Oman, Kuwait, Qatar, and Bahrain), were included. Studies were required to report on neonatal sepsis caused by common pathogens, specifically Klebsiella species, Coagulase-negative Staphylococcus species, *Staphylococcus aureus*, Streptococcus species, *Escherichia coli*, and Pseudomonas species, isolated from clinical samples (blood/CSF/urine, etc.) and diagnosed using standard laboratory methods (e.g., blood culture) with supporting clinical presentations. Neonates were defined as infants up to 3 months of age.

Studies were excluded if neonate patients could not be distinctly differentiated from older children or adults, if the study population was not based in the GCC countries, or if studies exhibited methodological deficiencies or lacked clear diagnostics criteria and clinical presentations.

### 2.3. Study Selection and Data Extraction

Articles identified by the search were imported and screened for duplication. Duplicate studies were excluded from the initial screening phase process. Titles and abstracts were screened to identify articles aligned with the research objectives. Full-text articles meeting the inclusion criteria were then selected for data extraction. The extracted data included: authors’ names, publication year, study setting (institution and country), study design (retrospective, cross-sectional), and sample size.

### 2.4. Quality Assessment Tool Used (MINORS)

The Methodological Index for Non-Randomized Studies (MINORS) tool was used to evaluate the methodological quality of all included studies. This validated instrument is specifically designed to assess non-randomized and observational research, focusing on key domains that reflect the rigor of study design, potential sources of bias, and overall methodological transparency.

The MINORS tool comprises eight domains applicable to non-comparative studies. Each domain is scored on a three-point scale, where a score of 0 indicates the item was not reported, 1 indicates it was reported but inadequately addressed, and 2 signifies that it was reported and adequately fulfilled. The assessed domains include a clearly stated aim, inclusion of consecutive patients, prospective collection of data, endpoints appropriate to the study’s objectives, unbiased assessment of endpoints, an appropriate follow-up period, a loss-to-follow-up rate of less than 5%, and a prospective calculation of study size.

All included studies were independently evaluated by two reviewers, and any discrepancies were resolved through discussion or consultation with a third reviewer. Each study received a total score ranging from 0 to 16, with higher scores reflecting stronger methodological quality and a lower risk of bias.

### 2.5. Statistical Analysis

The data extraction and screening were performed by using Rayyan software for systematic review, applying the defined inclusion and exclusion criteria. The four reviewers independently filtered search results, with a fifth reviewer available to resolve any disagreements. Also, the templates in Rayyan for systematic review were utilized to extract data from eligible research. Once the data was extracted, the reviewers organized the information into tables. Once screening has been done, all the included articles were analysed by Microsoft Excel version 2.91.24111439.

The included studies had significant differences in study designs, populations, and treatment methodologies, making the data heterogeneous and not appropriate to use in quantitative synthesis. Thus, this review was conducted without a meta-analysis. Rather, a systematic review was done to qualitatively summarize and interpret the pathogen profiles and antimicrobial resistance patterns of neonatal sepsis in the Gulf Cooperation Council (GCC).

## 3. Results

### 3.1. Characteristics of the Included Studies

**Figure 1:**
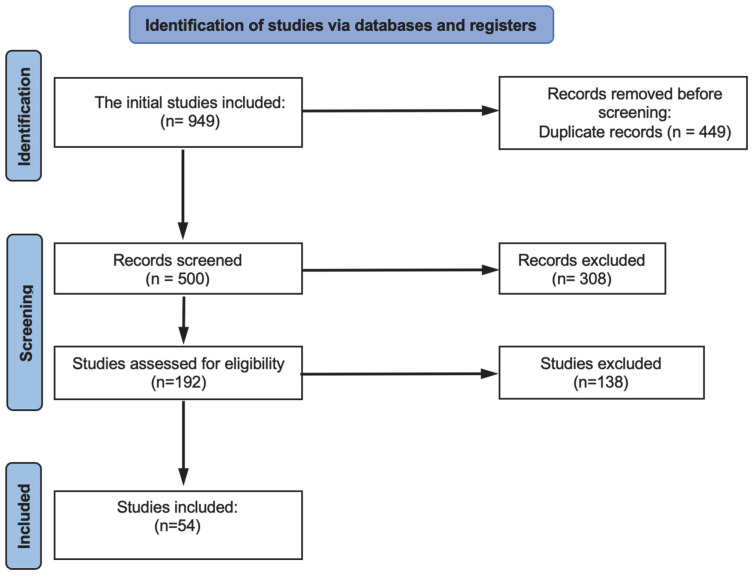
Flowchart (PRISMA) of the literature search and study selection process.

The study selection process, guided by PRISMA guidelines, is summarized in Figure 1. The initial database search yielded 949 titles. After removing duplicates, 500 studies underwent title and abstract screening to assess their potential relevance. Following the eligibility criteria, 192 full-text articles were retrieved for detailed evaluation. A total of 138 articles were excluded based on pre-defined exclusion criteria, resulting in a final inclusion of 54 articles that met the eligibility criteria [12,13,14,15,16,17,18,19,20,21,22,23,24,25,26,27,28,29,30,31,32,33,34,35,36,37,38,39,40,41,42,43,44,45,46,47,48,49,50,51,52,53,54,55,56,57,58,59,60,61,62,63,64,65].

A detailed summary of the methodological quality assessment of all included studies, based on the MINORS tool, is presented in Supplementary Table S1. The total MINORS scores among the 54 included studies ranged between 6/16 and 13/16, reflecting consistent methodological approaches and acceptable quality standards across the included literature. Studies that achieved higher scores commonly provided a clearly stated aim, appropriate endpoints, and adequate follow-up, while lower scores were primarily related to incomplete reporting of prospective data collection or sample size estimation.

These studies were published between 1983 and 2025 and conducted in the Arab Gulf countries, including Saudi Arabia, UAE, Oman, Kuwait, Qatar, and Bahrain. Across this timeframe, the GCC region experienced steady population growth and high birth rates, reflecting the expansion of neonatal care capacity across the Gulf countries. These births form the background population from which the 5177 neonatal isolates were derived, representing a comprehensive overview of sepsis trends over four decades of neonatal care improvement in the region [12,13,14,15,16,17,18,19,20,21,22,23,24,25,26,27,28,29,30,31,32,33,34,35,36,37,38,39,40,41,42,43,44,45,46,47,48,49,50,51,52,53,54,55,56,57,58,59,60,61,62,63,64,65] (Supplementary Table S2).

### 3.2. Distribution of the Pathogen Causing Neonatal Sepsis

**Figure 2:**
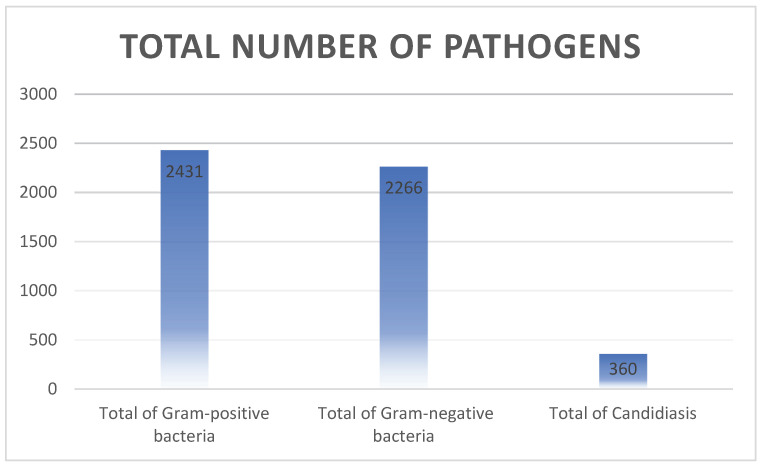
The proportion of species isolated from the blood of NICU patients.

A total of 5177 pathogens were isolated and subjected to antimicrobial susceptibility testing. Gram-positive bacteria accounted for 2495 (48%) of the isolates, while Gram-negative bacteria comprised 2318 (45%). Fungal isolates, predominantly Candida species, constituted the remaining 364 (7%) (Figure 2).

#### 3.2.1. Gram-Positive

**Figure 3:**
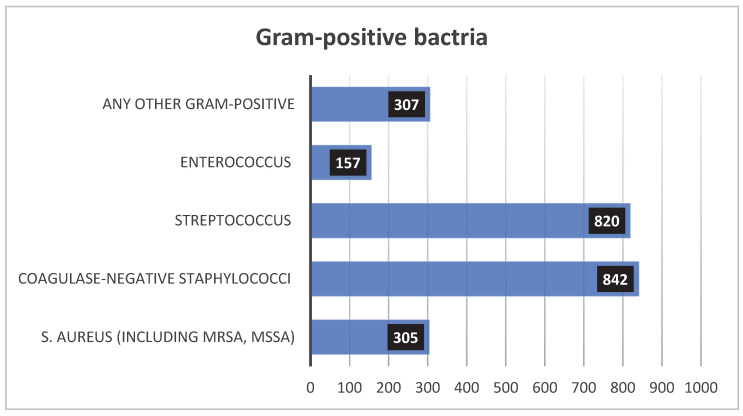
The proportion of Gram-positive bacterial species isolated from the blood of NICU patients.

Among the Gram-positive isolates, coagulase-negative Staphylococcus (CoNS) was the most frequently isolated organism, accounting for 901 isolates (17%), followed by Streptococcus species (primarily GBS) with 817 isolates (16%), *Staphylococcus aureus* with 309 isolates (6%), Enterococcus species with 161 isolates (3%), and other Gram-positive bacteria accounting for 307 isolates (6%) (Figure 3).

#### 3.2.2. Gram-Negative

**Figure 4:**
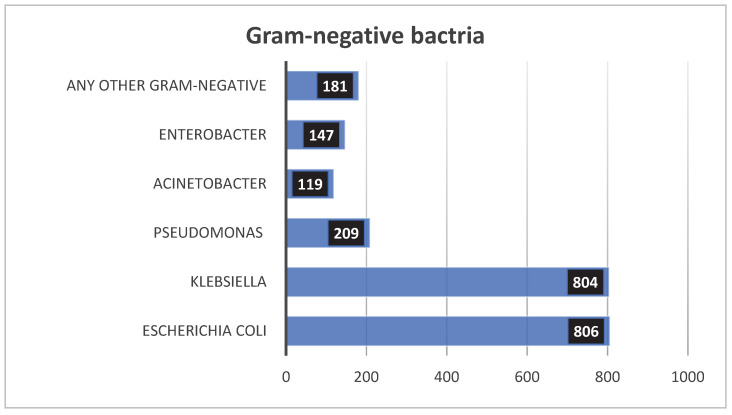
The proportion of Gram-negative bacterial species isolated from the blood of NICU patients.

In the Gram-negative group, Klebsiella species were the most common, with 825 isolates (16%), closely followed by *Escherichia coli* with 815 isolates (16%). Other Gram-negative isolates included Pseudomonas species (216 isolates, 4%), Enterobacter species (147 isolates, 3%), Acinetobacter species (121 isolates, 2%), and other Gram-negative bacteria (194 isolates, 4%). The remaining isolates were fungi, predominantly Candida species (364 isolates, 7%) (Figure 4).

#### 3.2.3. Leading Pathogens of Neonatal Sepsis

Coagulase-negative staphylococci and *Klebsiella* spp. were the leading pathogens in neonatal sepsis, accounting for 17% and 16% of cases, respectively.

### 3.3. Antimicrobial Susceptibility and Resistance Pattern in Gram-Positive Bacteria

**Figure 5:**
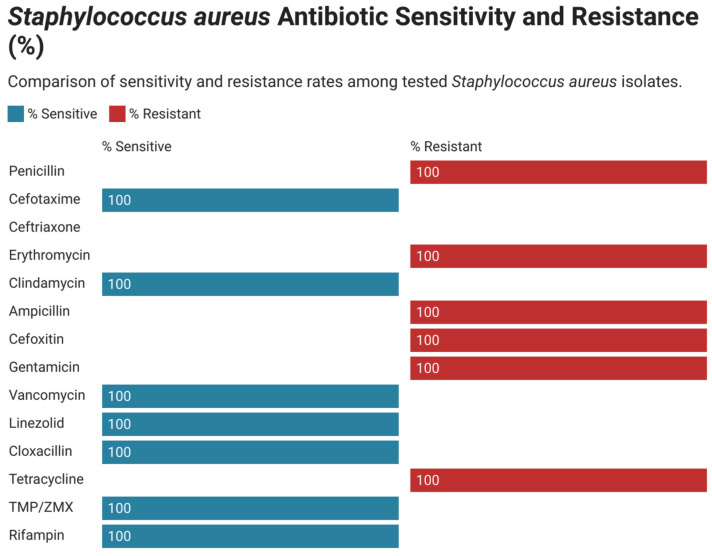
Antimicrobial resistance of all isolated *Staphylococcus aureus*.

**Figure 6:**
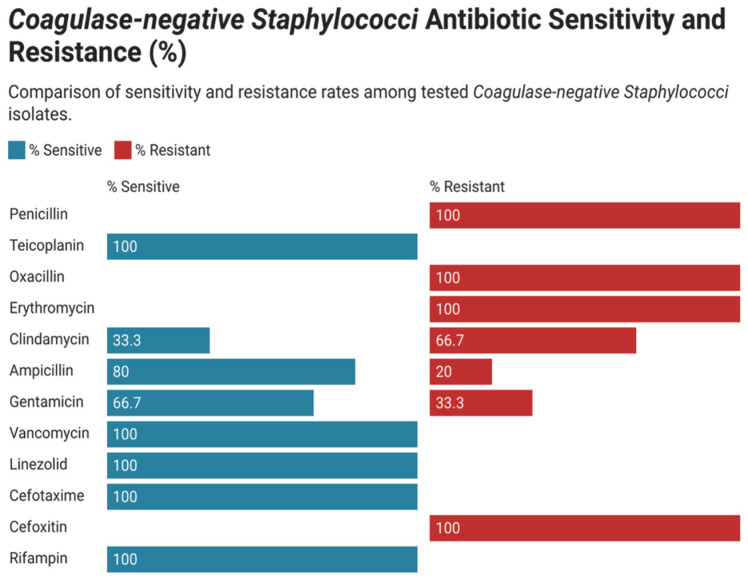
Antimicrobial resistance of all isolated Coagulase-negative Staphylococci.

**Figure 7:**
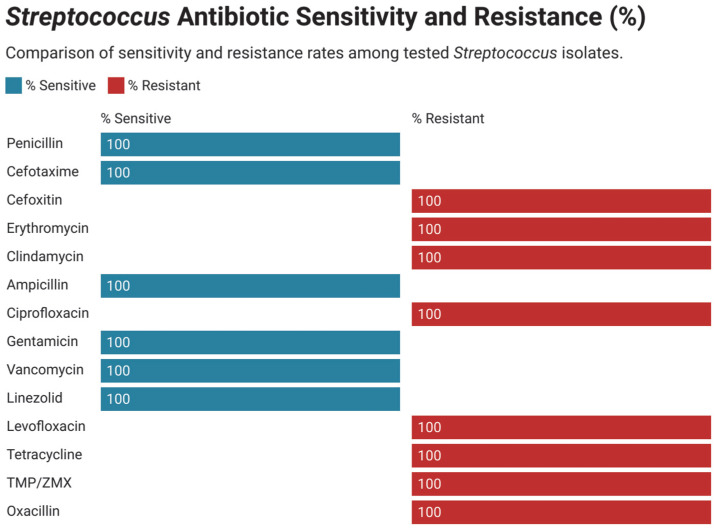
Antimicrobial resistance of all isolated Streptococcus species.

Antimicrobial susceptibility patterns of the frequently distributed bacterial isolates in neonatal intensive care units (NICUs) were assessed systematically. As shown in Figure 5, coagulase-negative Staphylococcus (CoNS) showed high rates of linezolid, vancomycin, and rifampin susceptibility in all studies and notable resistance toward erythromycin and penicillin. The profile of antimicrobial resistance of Streptococcus species (Figure 6) revealed a high resistance of erythromycin, tetracycline, clindamycin, and levofloxacin. Contrastingly, these isolates were mostly susceptible to ampicillin, vancomycin, linezolid, and cefotaxime. The *Staphylococcus aureus* isolates also showed great resistance to penicillin, oxacillin, erythromycin, as well as cefoxitin (Figure 7). It was however susceptible to linezolid, vancomycin, rifampin, cefotaxime. Finally, the Enterococcus species were found to be resistant mainly to gentamicin, erythromycin and tetracycline.

### 3.4. Antimicrobial Susceptibility and Resistance Pattern in Gram-Negative Bacteria

**Figure 8:**
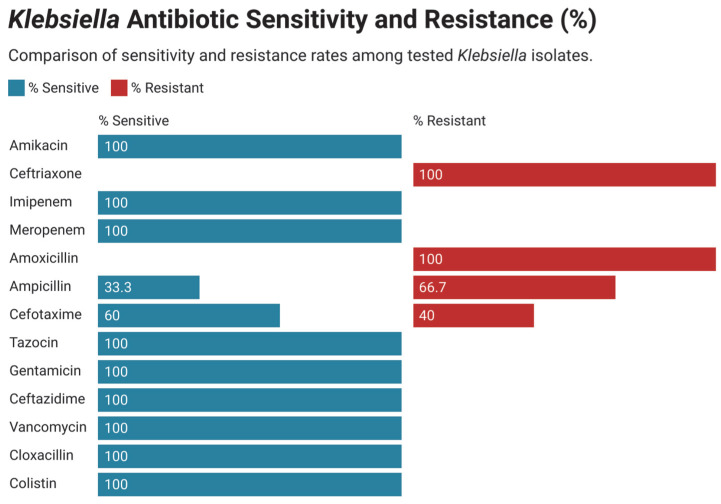
Antimicrobial resistance of all Klebsiella species.

**Figure 9:**
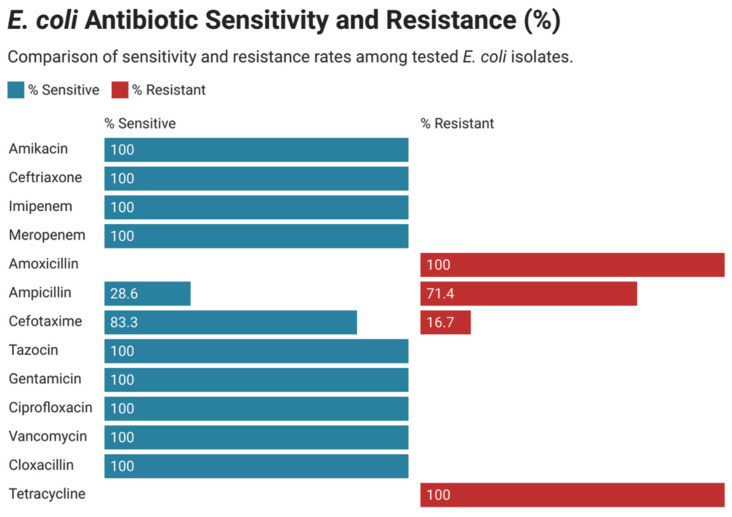
Antimicrobial resistance of all isolated *Escherichia coli*.

Among the Gram-negative isolates, Klebsiella species were the most frequently encountered and exhibited high levels of resistance to ampicillin, amoxicillin, levofloxacin, and ceftriaxone. However, they retained susceptibility to gentamicin, amikacin, cloxacillin, and colistin (Figure 8). *Escherichia coli* was the second most prevalent Gram-negative organism, demonstrating resistance to ampicillin, amoxicillin, cefotaxime, and tetracycline, while showing preserved sensitivity to gentamicin and amikacin (Figure 9).

Amikacin, gentamicin, and ciprofloxacin were active against other Gram-negative organisms, including *Pseudomonas* spp. Equally, gentamicin and amikacin were shown to be susceptible to *Acinetobacter* spp. On the other hand, *Enterobacter* spp. was mostly resistant to cefotaxime. It is noteworthy that carbapenems, meropenem, and imipenem were sensitive toward all the Gram-negative isolates in this study.

A total of 364 fungal isolates (7%) were identified, predominantly *Candida albicans* and *Candida parapsilosis*, with *Candida tropicalis* and *Candida glabrata* reported less frequently. Most isolates originated from blood and central venous catheter cultures. All species demonstrated high susceptibility to amphotericin B, fluconazole, and flucytosine, consistent across all six GCC countries. However, a subset of studies—particularly from Saudi Arabia and Kuwait—reported emerging fluconazole resistance among *C. tropicalis* and *C. glabrata*, highlighting the importance of routine antifungal susceptibility testing in neonatal intensive care units (NICUs).

### 3.5. Geographical Distribution of Neonatal Isolates in the GCC Region

The included studies originated from six Gulf Cooperation Council (GCC) countries—Saudi Arabia, Kuwait, the United Arab Emirates (UAE), Qatar, Oman, and Bahrain—representing a total of 5177 neonatal isolates obtained from 54 studies published between 1983 and 2025.

Saudi Arabia contributed the largest share, accounting for 2119 (40.9%) of all isolates, derived from 27 studies [12,13,14,15,16,18,19,22,24,25,26,27,28,31,35,39,40,42,44,45,48,50,51,52,54,63,64], including data from the multi-country collaboration by Hammoud et al. [20].

Kuwait ranked second, contributing 1815 isolates (35.1%) across 12 studies [21,29,30,36,37,46,47,56,57,61,62,65], which also incorporated the Kuwaiti arm of the same multi-country collaboration [20] and the largest single-country dataset in the region (*n* = 949) from Hammoud et al. [56].

The United Arab Emirates (UAE) accounted for 378 isolates (7.3%) from five studies [17,38,41,53,58], which likewise included the UAE subset reported by Hammoud et al. [20].

Qatar contributed 291 isolates (5.6%) across four studies [23,32,34,59], primarily hospital-based observational reports.

Oman accounted for 239 isolates (4.6%), derived from three studies [33,43,55].

Bahrain contributed 335 isolates (6.5%) from two studies [49,60], dominated by the large retrospective dataset from Bindayna et al. [49].

**Figure 10:**
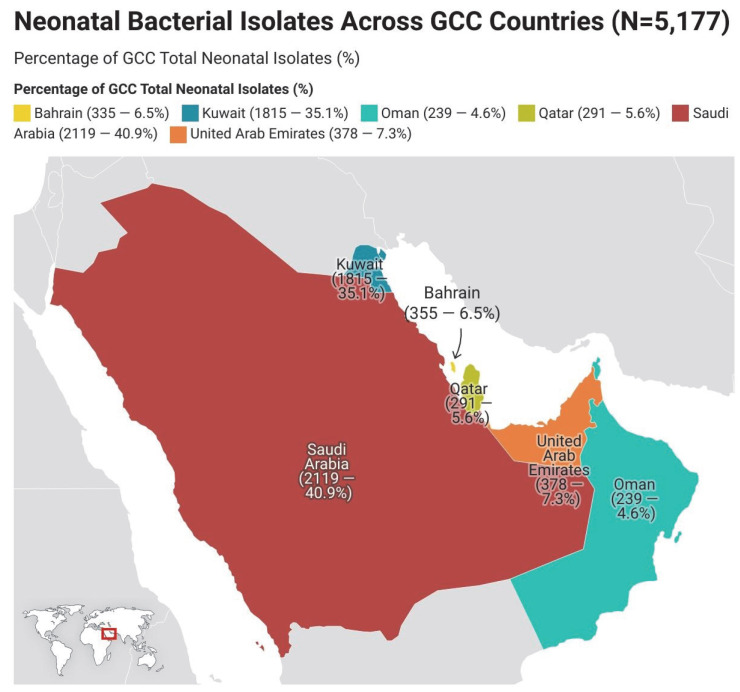
Neonatal Bacterial Isolates Across GCC Countries (N = 5177).

Overall, Saudi Arabia and Kuwait together represented more than three-quarters of all neonatal sepsis isolates reported in the GCC, reflecting their advanced neonatal intensive care unit (NICU) networks and active surveillance systems. The UAE, Qatar, Oman, and Bahrain provided smaller but valuable national datasets that enriched the regional representation. Collectively, these findings highlight a Saudi Kuwaiti leadership in neonatal sepsis research across the GCC and provide a robust foundation for future comparative analyses of antimicrobial resistance trends and neonatal infection-control strategies throughout the Arabian Gulf, (Figure 10).

## 4. Discussion

We analyzed data across all Gulf Cooperation Countries (GCCs) from 1983 onwards, providing a comprehensive overview of the pathogens causing neonatal sepsis and their antimicrobial resistance patterns. This study will serve as an essential foundation for future research to build on. Our main aim of this review is to evaluate the pathogenic profile of organisms causing neonatal sepsis and their antimicrobial resistance patterns in the GCC region. Neonatal sepsis burden is compounded by significant demographic shifts. The total fertility rate in the GCC has declined sharply, falling to 2.7 in Oman, 2.4 in Saudi Arabia, and below 2.0 in all other member states by 2022. This demographic trend underscores the critical public health importance of preventing neonatal mortality from sepsis, making each successful outcome more vital [66].

We included 54 studies in the current review. We identified a total of 5177 isolates, distributed as follows: 2495 g-positive bacteria, 2318 g-negative bacteria, and 364 fungi—primarily Candida species. Coagulase-negative staphylococcus (CoNS) was the major Gram-positive isolate, followed by Group B streptococcus (GBS) and *Staphylococcus aureus* (*S. aureus*), respectively. Among Gram-negative isolates, Klebsiella species were the most common (*n* = 825), closely followed by *E. coli* (*n* = 815). We found a trend for resistance to erythromycin and penicillin among Gram-positive organisms; however, most isolates remained sensitive to linezolid, vancomycin, and rifampin. All Gram-negative bacteria were susceptible to carbapenems, alongside gentamicin and amikacin.

Globally, the most common organism causing EOS is GBS [67,68]. In contrast, we found CoNS to be the most common Gram-positive pathogen, highlighting regional epidemiological differences. In the GCC and Middle East (ME) regions, our pathogen distribution and antimicrobial susceptibility are broadly consistent with previous meta-analyses [69,70]. However, unlike the meta-analysis in the GCC region done by Muhammad et al., in which they reported Staphylococcus species and GBS being the second and third most common isolated bacteria, respectively [69]. In contrast, we found Streptococcus species and *S. aureus* to be the second and third most common pathogens, respectively. Additionally, they found that *E. coli* was the most common Gram-negative isolate, contrasting the Klebsiella predominance in our review [69]. This minor difference is likely due to the number of studies and the inclusion of earlier cohorts in this review, which may suggest a historical shift in the pathogenic profile. This is especially true for GBS, in which healthcare advancement in antenatal vaginal and rectal screening for GBS colonization, followed by intrapartum antibiotic prophylaxis—where appropriate—with penicillin for colonized women has led to around an 85% reduction in neonatal sepsis secondary to GBS [67,68]. Thus, older cohorts may not reflect the current pathogenic profile. A proper solution to reduce the burden of sepsis is to develop and implement new screening and prophylaxis protocols after robust epidemiological profiling of local pathogen distribution. As such, consistent reporting of new cases, pathogenic profiles, and antimicrobial resistance patterns across hospitals is crucial for developing regional guidelines tailored to the local epidemiological data. We found that 7% of the cases of neonatal sepsis were due to Candida species. Candida infections vary across countries, possibly due to the antifungal prevention measures applied locally, evaluation of risk factors for fungal infection (low birth weight), and the lack of a sepsis scoring model for neonatal sepsis [71,72]. A recent systematic review of 476 neonatal cases by Sokou et al. showed prematurity, total parenteral nutrition, central line catheters, and perhaps the most important prior broad-spectrum antibiotic use to be associated with *C. auris* infection [73]. Both Ashkenazi-Hoffnung et al. and Sokou et al. showed concerning mortality rates among neonatal population, reaching up to 42% [73,74].

Concerning the antimicrobial resistance patterns, we noted a trend for penicillin resistance among Gram-positive organisms—CoNS and *S. aureus.* Contrasting with what we found, recent meta-analyses in the GCC and ME regions reported high susceptibility to these drugs; however, a recent review by Vogiantzi et al. from six Asian countries revealed that resistance to ampicillin, penicillin, and gentamicin is emerging among Gram-positive bacteria, consistent with our observation [69,70,75]. This is concerning given that CoNS were the most common isolates in our review, and the emerging resistance to penicillin could lead to carbapenems dependence as seen in lower-income countries. Thus, developing targeted antenatal screening protocols and awareness campaigns about vertical transmission risk could help reduce the incidence of such infections. Consequently, this will lower the dependence on carbapenems. Tight regulation on sterilization protocols is also of great importance and could help lower the incidence of sepsis, as the use of forceps during delivery has been linked to developing EOS [76]. Important to note, CoNS identification in culture for the first time is often indicative of contamination rather than true infection [76]. Therefore, studies should report the number of positive cultures in such cases; we highly encourage interpreting these results with caution. On the other hand, we observed a resistance to third-generation cephalosporins (3GC) among Gram-negative organisms, contrasting with Muhammad et al., in which they found susceptibility rates of 75% and 54.6% for *E. coli* and Klebsiella species, respectively [69]. In a recent meta-analysis in the ME region, the authors stratified ME countries into middle-income and high-income [70]. Notably, our results are similar to their pooled susceptibility rates of middle-income countries, despite the GCC being mostly high-income countries [70]. However, high-income countries in the ME region showed excellent susceptibility rates for 3GC [70]. Regarding fungal infection, Sokou et al.’s meta-analysis showed 97.4% resistance to fluconazole; they also showed a 67.1% resistance to amphotericin B, a cornerstone of treatment for invasive fungal disease [73]. Our result largely contradicts what Sokou et al. found; while the majority of our studies showed a high susceptibility to amphotericin B and fluconazole, we noted an emerging resistance trend toward these drugs—especially in Saudi Arabia and Kuwait [73]. These discrepancies could be explained by the time variation between the two reviews and region-specific resistance rates. Nevertheless, fungal infections—especially *C. auris*—pose a significant risk worldwide with high mortality, reaching up to 42% [73]. This necessitates an urgent, region-wide surveillance of fungal infection, especially in high-risk populations. These prompting data could be explained by the lack of antibiotic stewardship programs, widespread over-the-counter antibiotic use, and inadequate authority regulation on screening protocols in the regional guidelines. Interestingly, the Swiss Society of Neonatology recently changed its guidelines on the evaluation of early-onset sepsis (EOS), switching to a probabilistic approach by not recommending the use of EOS calculators due to their low sepsis incidence [77]. They proposed this approach in the efforts to lower antibiotic use and help improve the susceptibility rates locally [77]. Future regional guidelines could discuss similar approaches, after sufficient reporting of sepsis incidence locally, to help decrease antibiotic overuse.

The American Academy of Pediatrics (AAP) currently recommends a combination of ampicillin and aminoglycoside as initial empirical therapy until culture results are available [78,79]. MOH in Saudi Arabia advocates for the same approach with their guidelines on EOS diagnosis and treatment [80]. Our results still support this combination, but with caution on emerging resistance to penicillin—especially in low- and middle-income countries [69]. The non-trivial candidal isolates found in our review highlight the need for strict regional monitoring of antifungal prevention measures. Local authorities should consider amphotericin B as an empirical regimen if fungal infection is common [72,81]. However, we observed heterogeneity in clinical practice guidelines across the GCC. While Saudi Arabia and Qatar have implemented specific, region-wide directives, other countries appear to rely on an institutional approach rather than regional guidance [80,82,83,84,85]. Additionally, there is a lack of official, national-level guidelines for LOS across the GCC [86]. Implementing a standardized framework for reporting antimicrobial resistance patterns across the GCC will help to better understand the current burden and develop a specified treatment approach for local resistance patterns.

Several GCC nations have moved beyond emphasizing antibiotic stewardship programs (ASP) to making them a regulatory requirement. The United Arab Emirates (UAE) is a regional leader in this matter, mandating the implementation of ASP across all healthcare facilities [87]. Establishment of a neonatal-specified ASP in low- and middle-income countries is especially important, as it has been shown in a Lebanese study to lead to a 35% reduction in antimicrobial usage within the first three months, with a median decline of 63% over five years [88]. Among the most prescribed empiric therapies, ampicillin and gentamicin, this program led to a decrease in their usage by 63% and 79%, respectively [88]. Crucially, this significant reduction did not lead to evidence of negative impact on patients’ outcomes [88]. A study conducted in Dubai NICU provided evidence linking the stewardship implementation to a notable decline in sepsis cases caused by *Candida* and ESBL-producing *Klebsiella*, specifically restricting the use of third-generation cephalosporins and glycopeptides [89]. In accordance with Saudi Arabia guidelines on EOH diagnosis and treatment, advise against the use of cefotaxime as an empiric treatment except in suspected meningitis cases [80]. This highlights the important role that these programs have by optimizing patient care by using appropriate antibiotics and by reducing antimicrobial resistance.

## 5. Strengths and Limitations

We believe that this systematic review is the only review focusing on neonatal sepsis incidence and antimicrobial resistance patterns in the GCC countries from a long-term point of view. By including all pathogens in the protocol of this review, we were able to identify 7% Candida isolates, highlighting the significance of fungal pathogens in the GCC region. Our large number of included articles will provide a robust foundation for future studies to build on to help reduce the neonatal sepsis burden in the GCC region. Nevertheless, we noted some limitations. First, we did not stratify early-onset and late-onset sepsis in the analysis. Second, risk factors and antimicrobial resistance patterns for multidrug-resistant isolates were not analyzed. Third, most of the included studies (>25 studies) were from Saudi Arabia, and ≤2 studies were in Bahrain and Oman, which provides uneven distribution and limits the generalizability of the results. Fourth, the lack of unified national protocols for several countries limited our interpretation of the current situation in the region; however, this is an inherent limitation in the regional healthcare infrastructure. Fifth, we acknowledge that some unpublished institutional protocols exist that we could not reach due to the inherent limitation of our design, which is based on published literature. Sixth, only a limited number of studies within the review specifically classified neonatal sepsis into early-onset (EOS) and late-onset (LOS) categories. Most included articles reported neonatal sepsis as a single entity without temporal subclassification. Therefore, a pooled analysis of EOS and LOS prevalence could not be accurately conducted across the GCC dataset.

## 6. Recommendations

Future studies should focus on the incidence of neonatal sepsis caused by multidrug-resistant organisms in the GCC region. Magnitude and risk factors for neonatal sepsis in the GCC regions are potential areas to explore. Development of unified, evidence-based national guidelines for both EOS and LOS in all GCC nations is of the utmost importance. Further surveillance studies to quantify the true prevalence of fungal infection, especially *C. auris,* in the GCC are warranted. Future studies in the GCC region should adopt standardized reporting that distinguishes between EOS and LOS to allow more detailed epidemiological comparisons.

## 7. Conclusions

Findings of this systematic review were that the most reported Gram-negative pathogens that caused neonatal sepsis in the Arab Gulf region were Klebsiella pneumoniae and *Escherichia coli*. Coagulase-negative Staphylococcus (CoNS) bacteria were the most common Gram-positive bacteria, followed by Streptococcus species, with the most likely *Streptococcus agalactiae* (Group B).

Amikacin, gentamicin, meropenem, and imipenem were most susceptible to Gram-negative pathogens, and vancomycin and linezolid were most susceptible to Gram-positive. The results point to the need for regional monitoring of the prevalence and antimicrobial resistance patterns of pathogens to inform the use of empirical treatment, both to target resistance patterns and enhance the survival of neonates in the GCC area.

Despite these insights having been made, the review also brought to light the fact that many of the Gulf countries did not provide adequate and current data. Thus, additional multicentric studies and strong national surveillance systems are imperative in determining the epidemiology and trends of antimicrobial resistance of neonatal sepsis within the GCC region.

## Data Availability

No new data were created or analyzed in this study. Data sharing is not applicable to this article.

## Supplementary Materials

The following supporting information can be downloaded at: https://www.mdpi.com/article/10.3390/children12111475/s1.
