# Supplementary material for: Pathogen Profiles and Antimicrobial Resistance Patterns of Neonatal Sepsis in the Gulf Cooperation Council: A Systematic Review
Source: Children (Basel). 2025 Nov 1;12(11):1475. doi: 10.3390/children12111475 (PMC12650816; doi:10.3390/children12111475)
Supplement: Supplementary file 1 [file children-12-01475-s001.zip › children-3895054-supplementary.pdf]

Supplementary Tables

Title: Pathogen Profiles and Antimicrobial Resistance Patterns of Neonatal Sepsis in the Gulf Cooperation Council: A Systematic Review

Table S1: The quality scores of the included studies assessed by the MINORS tool

Authors, year of publication	Clearly Stated Aim	Inclusion of Consecutive Patients	Prospective Collection of Data	Endpoints Appropriate to the Aim of the Study	Unbiased Assessment of the Study Endpoint	Follow-up Period Appropriate to the Aim of the Study	Loss to Follow-up < 5 %	Prospective Calculation of Study Size	Total (/16)
Talat et al., 2015 [12]	2	1	1	2	0	1	2	0	9/16
Ghaziaa, 2022 [13]	2	0	0	2	0	1	1	0	6/16
Khaloud et al., 2021 [14]	2	1	0	2	0	1	1	0	7/16
Azzah S. Alharbi, 2022 [15]	2	1	0	2	0	1	1	0	7/16
Elham Essa Bukhari & A. Alrabiaah, 2011 [16]	2	1	0	2	0	1	1	0	7/16
Carole et al., 2020 [17]	2	1	1	2	1	1	2	0	10/16
Hind et al., 2023 [18]	2	1	0	2	0	1	1	0	7/16
Mountasser et al., 2018 [19]	2	1	0	2	0	1	1	0	7/16
Majeda S. Hammoud et al., 2017 [20]	2	2	2	2	1	2	2	0	13/16

Majeda S. Hammoud et al., 2012 [21]	2	1	0	2	0	1	1	0	7/16
Abdulrahman S. Bazaid et al., 2023 [22]	2	1	0	2	0	1	1	0	7/16
Afif Ahmed et al., 2013 [23]	2	1	0	2	0	1	1	0	7/16
Maha Abd El Hafez et al., 2011 [24]	2	1	0	2	0	1	1	0	7/16
Abdulhadi Husun et al., 2017 [25]	2	1	0	2	0	1	1	0	7/16
Mohammed Almogbel et al., 2021 [26]	2	1	0	2	0	1	1	0	7/16
Khalid M. AlFaleh et al., 2010 [27]	2	1	0	2	0	1	1	0	7/16
Talat A. El-Kersh et al., 2016 [28]	2	1	1	2	0	1	2	0	9/16
Fatemah Al-Mutairi et al., 2018 [29]	2	1	0	2	0	1	1	0	7/16
Abdullah Al-Taiar et al., 2011 [30]	2	2	2	2	1	2	2	0	13/16
Abdulrahman S. Bazaid et al., 2022 [31]	2	1	0	2	0	1	1	0	7/16

Asmir Jonuzi et al., 2021 [32]	2	0	0	2	0	1	1	0	6/16
Bhaskar Gupta et al., 2010 [33]	2	1	0	2	0	1	1	0	7/16
Sajid Salim Thyvilayil et al., 2025 [34]	2	2	2	2	1	2	2	0	13/16
A. Dawodu et al., 1997 [35]	2	1	0	2	0	1	1	0	7/16
Adnan El-Kishawi et al., 2008 [36]	2	0	0	2	0	1	1	0	6/16
Eiman M. Mokaddas et al., 2011 [37]	2	2	2	2	1	2	2	0	13/16
Eman Al Reyami et al., 2009 [38]	2	1	0	2	0	1	1	0	7/16
Ali H. Almudeer et al., 2020 [39]	2	1	0	2	0	1	1	0	7/16
Abdulrahman Al-Matary et al., 2019 [40]	2	1	0	2	0	1	1	0	7/16
Mustafa AlAbdullatif et al., 2019 [41]	2	1	0	2	0	1	1	0	7/16
M. A. Fattah et al., 2017 [42]	2	1	0	2	0	1	1	0	7/16
Al-Masroori	2	1	0	2	0	1	1	0	7/16

et al., 2019 [43]									
Al-Rafiaah et al., 2016 [44]	2	1	0	2	0	1	1	0	7/16
Al-Zahrani et al., 2015 [45]	2	1	0	2	0	1	1	0	7/16
Al-Essa et al., 2000 [46]	2	1	0	2	0	1	1	0	7/16
Khan et al., 2019 [47]	2	1	0	2	0	1	1	0	7/16
Abu Srair et al., 2006 [48]	2	1	0	2	0	1	1	0	7/16
Bindayna et al., 2006 [49]	2	1	0	2	0	1	1	0	7/16
A. Asindi et al., 1999 [50]	2	1	0	2	0	1	1	0	7/16
A. Ohlsson et al., 1986 [51]	2	1	0	2	0	1	1	0	7/16
Ali M. Elbashier et al., 1994 [52]	2	1	0	2	0	1	1	0	7/16
Ayman Koutouby et al., 1995 [53]	2	1	0	2	0	1	1	0	7/16
Enas Sh. Khater et al., 2020 [54]	2	1	0	2	0	1	1	0	7/16
Mohamed Abdellatif et al., 2019 [55]	2	1	0	2	0	1	1	0	7/16
Majeda S. Hammoud et al., 2012 [56]	2	2	2	2	1	2	2	0	13/16

Aishah Al-Haqan et al., 2020 [57]	2	1	0	2	0	1	1	0	7/16
AlZuheiri et al., 2021 [58]	2	2	2	2	1	2	2	0	13/16
Shittu et al., 2021 [59]	2	0	0	2	0	1	1	0	6/16
Lulwa Al Mannaei et al., 2017 [60]	2	1	0	2	0	1	1	0	7/16
N. Al-Sweih et al., 2005 [61]	2	1	0	2	0	1	1	0	7/16
Noura Al-Sweih et al., 2008 [62]	2	1	0	2	0	1	1	0	7/16
El-Kersh A. Talat et al., 2012 [63]	2	1	0	2	0	1	1	0	7/16
Amr Mohamed et al., 2020 [64]	2	1	0	2	0	1	1	0	7/16
Fatemah Al-Mutairi et al., 2019 [65]	2	1	0	2	0	1			

Table S2. Neonatal sepsis pathogen profiles of the isolated pathogens in each included study.

Author's Name	Year	Study design	Location	Sample size	Isolated pathogens
Talat et al. [12]	2015	Observational study	Saudi Arabia	150 neonates	*E. coli, Klebsiella pneumoniae, Pseudomonas aeuroginosa*
Ghaziaa [13]	2022	Case report	Saudi Arabia	5 neonates	MDR *Klebsiella pneumoniae*
Khaloud, et al. [14]	2021	Observational study	Saudi Arabia	123 culutre proven cases out of 151 neonates	*Staphylococcus aureus, Streptococcus pyogenes, Klebsiella pneumoniae*
Azzah S. Alharbi, [15]	2022	Retrospective study	Saudi Arabia	40 culutre proven cases out of 246 neonates	Coagulase-negative Staphylococcus (CoNS) *Klebsiella spp., Streptococcus agalactiae*
Elham Essa Bukhari, Abdulkarim Abdullah Alrabiaah, [16]	2011	Retrospective study	Saudi Arabia	12 culutre proven cases out of 304 neonates	CoNS, *Staphylococcus aureus, Staphylococcus hominis*
Carole, et al. [17]	2020	Comparative study	UAE	61 culutre proven cases out of 1,813 rectal swabs	*Klebsiella pneumoniae, Escherichia coli, Pseudomonas aeruginosa*
Hind, et al. [18]	2023	Prospective study	Saudi Arabia	110 neonates	CoNS, *E. coli, Klebsiella pneumoniae*
Mountasser et al. [19]	2018	Retrospective study	Saudi Arabia	70 positive culutre out of 295 neonates	CoNS, Gram-negative Bacilli, *Staph. aureus*
Majeda S. Hammoud et al. [20]	2017	Observation prospective al study	Saudi Arabia, Kuwait, UAE	785 isolates (Saudi Arabia: 337 , Kuwait: 318 , UAE: 130.)	CoNS, Klebsiella spp, *E. coil*
Majeda S. Hammoud et al. [21]	2012	Retrospective study	Kuwait	89 neonates	Candida
Abdulrahman S. Bazaid et al. [22]	2023	retrospective	Saudi Arabia	51 neonates	Methicillin-resistant Staphylococcus aureus (MRSA), *K pneumoniea, Serratia*

AFIF AHMED et al, [23]	2013	Retrospective study	Qatar	176 neonates	66% gram +ve, 18% Fungi, 16% gram –ve.
Maha Abd El Hafez et al. [24]	2011	Retrospective study	Saudi Arabia	Maternal Cohort excluded	Methicillin resistant *Staphylococcus epidermidis*
Abdulhadi Husun et al. [25]	2017	case controlled study	Saudi Arabia	24 culutre proven cases out of 80 preterm neonates.	CoNS, S. *aureus, klebsiella Pneumonia*
Mohammed Almogbel et al. [26]	2021	Retrospective study	Saudi Arabia	72 culutre proven cases out of 821 preterm neonates.	*Klebsiella Pneumonia, E. coli*
Khalid M AlFaleh et al. [27]	2010	Retrospective study	Saudi Arabia	98 culutre proven cases out of 237 preterm neonates.	CoNS, *Klebsiella Pneumonia, E. coli*
Talat A. El-Kersh et al. [28]	2016	Prospective study	Saudi Arabia	Maternal Cohort excluded	*Enterococcus faecalis, Staphylococcus epidermidis, Staphylococcus aureus*
Fatemah Al-Mutairi et al. [29]	2018	Observational study	Kuwait	Maternal Cohort excluded	GBS AMR/susceptibility focus
Abdullah Al-Taiar et al. [30]	2011	Prospective study	Kuwait	153 neonates	GBS, *Klebsiella Pneumonia,* Coagulase-negative staphylococci
Abdulrahman S. Bazaid et al. [31]	2022	Retrospective study	Saudi Arabia	54	*Klebsiella pneumoniae, E. coli, Acinetobacter baumannii*
Asmir Jonuzi et al. [32]	2021	Case report	Qatar	1	*E. coli*
Bhaskar Gupta et al. [33]	2010	Retrospective study	Oman	71 culture-proven cases out of 2181	GBS, *Staphylococcus epidermidis*, Group D Enterococcus, *E. coli, Pseudomonas, Acinetobacter, Klebsiella,* and MRSA.
Sajid Salim Thyvilayil et al. [34]	2025	Retrospective study	Qatar	113 culture-proven cases out of 123,878	GBS

A Dawodu et al. [35]	1997	Case-control study	Saudi Arabia	61 culture-proven cases out of 1291	*Staphylococcus epidermidis, Klebsiella-Enterobacter-Serratia group, Pseudomonas aeruginosa*
Adnan El-Kishawi et al. [36]	2008	Case Report	Kuwait	1	*Streptococcus pneumoniae*
Eiman M Mokaddas et al. [37]	2011	Prospective study	Kuwait	78 culture-proven cases out of 3408	CoNS, *Pseudomonas aeruginosa, Klebsiella pneumoniae*
Eman Al Reyami et al. [38]	2009	Retrospective study	United Arab Emirates	1 ear-swab-proven case out of 239	MRSA
Ali H Almudeer et al. [39]	2020	Retrospective study	Saudi Arabia	126 culture-proven cases out of 28,337	*E. coli*, GBS, CoNS
Abdulrahman Al-Matary et al. [40]	2019	Retrospective study	Saudi Arabia	245 culture-proven cases	GBS, *E. coli, Staphylococcus spp., Klebsiella pneumoniae*
Mustafa AlAbdullatif et al. [41]	2019	Retrospective study	United Arab Emirates	80 culture-proven cases out of 2732	*Klebsiella pneumoniae, E. coli, Pseudomonas aeruginosa*
M. A. Fattah et al. [42]	2017	Cross-sectional	Saudi Arabia	Maternal Cohort excluded	*E. coli,* GBS, *Haemophilus influenza, Staphylococcus aureus*
Al-Masroori et al. [43]	2019	Retrospective study	Oman	43 culture-proven cases out of 83,000	GBS
Al-Rafiaah et al, [44]	2016	Cross-sectional	Saudi Arabia	43 culture-proven cases out of 85	CoNS, *E. coli, Pseudomonas aeruginosa, Staphylococcus aureus*
Al-Zahrani et al. [45]	2015	Prospective study	Saudi Arabia	25 culture-proven cases out of 100	Klebsiella spp., *E. coli*, GBS, CoNS
Al-Essa et al. [46]	2000	Cohort study	Kuwait	Maternal Cohort excluded	*Candida albicans, Candida parapsilosis, Candida tropicalis*
Khan et al. [47]	2019	Retrospective study	Kuwait	134 culture-proven	*Candida lusitania,*

				cases out of 7068	
Abu Srair et al. [48]	2006	Prospective study	Saudi Arabia	53 neonates	GBS, *E. coli, Sterptococcus faecalis.*
Bindayna et al. [49]	2006	Retrospective study	Bahrain	335 isolates	CoNS, *E. coli,* GBS ,
A. Asindi et al. [50]	1999	Prospective study	Saudi Arabia	106	*E. coli, Klebsiella pneumonia, Staphylococcus aureus*
A. Ohlsson et al. [51]	1986	Retrospective study	Saudi Arabia	50	*E. coli, Klebsiella pneumonia, Staphylococcus aureus*
Ali M. Elbashier et al. [52]	1994	Retrospective study	Saudi Arabia	144	*E. coli, Klebsiella pneumonia, Pseudomonas aeruginosa*
Ayman Koutouby et al. [53]	1995	Retrospective study	United Arab Emirates	106	GBS, *E. coli, Staphylococcus epidermidis*
Enas Sh. Khater et al. [54]	2020	Cross sectional	Saudi Arabia	120	CoNS, E. *coli, Staphylococcus aureus*
Mohamed Abdellatif et al. [55]	2019	Cross sectional	Oman	125	CoNS, *Klebsiella pneumonia, E. coli,*
Majeda S. Hammoud et al. [56]	2012	Prospective study	Kuwait	949	CoNS, *Klebsiella pneumonia, E. coli,*
Aishah Al-Haqan et al. [57]	2020	Retrospective study	Kuwait	93	*Staphylococcus epidermidis, Staphylococcus capitis, Staphylococcus hominis*
AlZuheiri et al. [58]	2021	Prospective study	United Arab Emirates	Maternal Cohort excluded	GBS AMR/susceptibility focus
Shittu et al. [59]	2021	Case report	Qatar	1	*E. coli*
Lulwa Al Mannaei et al. [60]	2017	Retrospective study	Bahrain	Maternal Cohort excluded	CoNS
N. Al-Sweih et al. [61]	2005	Descriptive observational study	Kuwait	Maternal Cohort excluded	GBS AMR/susceptibility focus
Noura Al-Sweih et al. [62]	2008	Retrospective study	Kuwait	Maternal Cohort excluded	*Candida parapsilosis, C. albicans*

El-kersh A. Talat et al. [63]	2012	Observational study	Saudi Arabia	Maternal Cohort excluded	GBS AMR/susceptibility focus
Amr Mohamed et al. [64]	2020	Cross-sectional study	Saudi Arabia	Maternal Cohort excluded	GBS AMR/susceptibility focus
Fatemah Al-Mutairi et al. REPEATED 2 times [65]	2019	Cross-sectional study	Kuwait	Maternal Cohort excluded	GBS AMR/susceptibility focus

Abbreviations: CoNS – Coagulase-negative staphylococci; GBS – Group B streptococcus (Streptococcus agalactiae); KES group – Klebsiella-Enterobacter-Serratia group; MRSA – Methicillin-resistant Staphylococcus aureus; AMR – Antimicrobial resistance; ESBL – Extended-spectrum β-lactamase; MDR, multidrug-resistant; UAE – United Arab Emirates; RAK – Ras Al Khaimah; GCC – Gulf Cooperation Council.
